# Foliar mineral nutrient uptake in carnivorous plants: what do we know and what should we know?

**DOI:** 10.3389/fpls.2013.00010

**Published:** 2013-01-31

**Authors:** Lubomír Adamec

**Affiliations:** Department of Functional Ecology, Section of Plant Ecology, Institute of Botany of the Academy of Sciences of the Czech RepublicTřeboň, Czech Republic

## Carnivorous plants as ecological group

Carnivorous plants (CPs) usually grow in nutrient-poor, wet or aquatic environments and possess foliar traps which capture animal prey (Juniper et al., 1989). There are about 600 terrestrial and 50 aquatic or amphibious species of CPs which supplement the conventional mineral nutrient uptake by roots or shoots from their environment by the absorption of nutrients (mainly N, P, K, Mg) from prey carcasses captured by their traps (for the review, see Adamec, 1997, 2002, 2011a). Among vascular plants, they probably have the greatest capacity of foliar mineral nutrient uptake which can cover 5–100% of their seasonal N and P gain (consumption) but only 1–16% for K from captured prey (Adamec, 1997, 2011a). The main ecophysiological strategy of terrestrial species as S-strategists is slow growth and very effective mineral nutrient economy. Due to new discoveries (e.g., Spomer, 1999; Anderson and Midgley, 2003; Pavlovič, 2012), the boundary between carnivory and non-carnivory remains slightly blurred. In line with recent findings, the concept of plant carnivory should be defined with an emphasis on the main benefit of carnivory—the uptake of mineral nutrients from prey (directly or indirectly) captured by traps. Moreover, as all plants with glandular hairs are potentially carnivorous (Spomer, 1999), a defining statement that foliar nutrient uptake from prey must be “ecologically significant” for CPs seems reasonable (Płachno et al., 2009) whereas criteria such as producing their own digestive enzymes or prey attraction are only marginal.

## Stimulation of root mineral nutrient uptake by foliar nutrient uptake

The four principal processes that determine the mineral nutrient budget in terrestrial CPs are: foliar nutrient uptake from prey, root nutrient uptake from the soil, mineral nutrient reutilization from senescing shoots and stimulation of root nutrient uptake by foliar nutrient uptake. This stimulated uptake was repeatedly confirmed in about 10 terrestrial species under greenhouse or field conditions (e.g., Hanslin and Karlsson, 1996; Adamec, 1997, 2002, 2011a) and this presumably represents one of the most important ecophysiological adaptations of CPs. Generally, CPs fed on insects or mineral nutrient solutions grew rapidly and accumulated far more mineral nutrients in their total produced biomass (about 1.6-27 × more for N, P, K, Ca, and Mg compared to unfed control plants) than they could theoretically take up from the limited foliar nutrient supply. Thus, mineral substances taken up by leaves from prey stimulated, in an unknown way, the activity of the roots which then took up the quantity of nutrients needed for increased growth from the mineral-poor soil. It is fascinating that the stimulated uptake is to a much greater physiological extent than the direct uptake of nutrients from prey itself. It is possible to assume that the extent of this stimulation will be several times greater for K, Ca, and Mg uptake than that for N and P under natural conditions as prey are a rather poor source of these metallic cations. The essence of the stimulation of root uptake in CPs has not yet been explained. A stepwise feeding on prey in the field revealed that the stimulatory effect was of a quantitative nature, dependent on the amount of prey (Hanslin and Karlsson, 1996). In three *Drosera* species, slightly greater root lengths could only explain about 17% of the uptake stimulation, the higher theoretical uptake rate of roots per unit root biomass being only about 15–30%, but the greater root biomass could explain up to 70–85% of the effect (Adamec, 2002). Root aerobic respiration, however, was unchanged. Moreover, the stimulatory effect on the roots did not correlate with tissue mineral nutrient content in the roots or shoots and the root: shoot biomass ratio of fed plants slightly decreased. Phosphate alone could cause the stimulation (Karlsson and Carlsson, 1984) but the role of other nutrients (especially N) is as yet unknown.

The explanation of the stimulatory effect on the roots should be a priority challenge for CP ecophysiologists. As shown by Adamec (2002), the effect could not be caused by an increased root mineral nutrient content. Evidently, one of the possible mechanisms of the root uptake stimulation could be based on an increased photosynthetic rate in leaves and subsequent allocation of photosynthates to roots. Nevertheless, stimulation of CP photosynthesis by feeding on prey is still a great issue. While an increased photosynthetic rate due to prey feeding has recently been proven only in *Sarracenia* and *Nepenthes* species with pitcher traps (Farnsworth and Ellison, 2008; Pavlovič et al., 2009), no increase occurred in *Drosera* or *Pinguicula* species (Méndez and Karlsson, 1999) though commonly these latter two genera exhibit marked root uptake stimulation (Adamec, 1997, 2002, 2011a). It is thus possible that the photosynthetic response to prey feeding is genus specific. Another direction of the stimulation study could be hormonal: to investigate the distribution of cytokinins and the strength of the sinks within CP roots. Finally, modern transcriptomic research should reveal gene families which are switched on or off in stimulated and control roots, respectively (*sensu* Ibarra-Laclette et al., 2011). In any case, direct measurements of mineral nutrient (ammonium, phosphate, K^+^) uptake by intact or excised CP roots are essential for quantifying the root uptake affinity and achieving any progress in this field. On the other hand, following from numerous data (Adamec, 1997, 2002, 2011a; Moran et al., 2010), the affinity of mineral nutrient uptake from prey by traps is rather high.

## Foliar nutrient uptake in aquatic carnivorous plants

Aquatic carnivorous plants (ACPs) comprise the species *Aldrovanda vesiculosa* (Droseraceae) and about 50 species of the genus *Utricularia* (Lentibulariaceae). They usually grow in shallow, standing humic waters which are usually poor in N and P (Adamec, 1997, 2011a). ACPs are ecophysiologically quite dissimilar to their terrestrial counterparts; they take up all necessary nutrients either directly from the water by their shoots or from animal prey by traps. Their entirely rootless shoots are mostly linear and, under favourable conditions even in barren habitats, they exhibit very rapid apical shoot growth of 3–4 leaf nodes d^−1^ while their shoot bases decay at this same high rate. The very rapid growth requires a combination of several ecophysiological processes including the capture of animal prey, very high photosynthetic rates, very efficient mineral nutrient uptake from water and efficient mineral nutrient reutilization (except K^+^) from senescent shoots (Adamec, 1997, 2011a).

Foliar nutrient uptake in ACPs is still veiled in mystery caused by methodical difficulties. Unlike terrestrial CPs, in which the efficiency of foliar mineral uptake from prey has been quantified for several nutrients (Adamec, 2002, 2011a; Płachno et al., 2009), such information is almost entirely lacking for aquatic CPs. Friday and Quarmby (1994) estimated an 83% efficiency of N uptake from model mosquito larvae in *Utricularia vulgaris* traps which is higher than that in terrestrial species (cf. Adamec, 2011a). Although prey feeding markedly enhanced the growth of aquatic CPs in many growth experiments (Adamec, 1997, 2011a), the uptake efficiency from prey is unknown. Moreover, the available results do not allow one to determine whether captured prey leads to a stimulation of nutrient uptake by shoots. However, it is possible to deduce from the growth in very oligotrophic waters with zero prey availability that aquatic *Utricularia* species have a very high shoot uptake affinity for mineral nutrients: at least 0.4 μM for NH^+^_4_ and 0.1 μM for phosphate (see Adamec, 2009). Again, the affinity has never been measured.

One of the challenges for study is K^+^ economy in aquatic CPs. Rapidly growing aquatic species exhibit a high shoot K^+^ content of 1.5–5% of dry weight but always lose *all* K^+^ in their senescent shoots (but very effectively re-utilize N and P) which greatly contrasts with very efficient K^+^ reutilization in terrestrial CPs (Adamec, 1997, 2011a). As prey is a relatively poor K^+^ source, aquatic CPs must rely mainly on permanent rapid K^+^ uptake from the ambient water by shoots. In *A. vesiculosa*, surprisingly, K^+^ was taken up only by basal shoot segments, but not apical ones which permanently need much K^+^ for their rapid apical growth (Adamec, 2000). Presumably, K^+^ is allocated from basal shoot segments to the apical ones. Repeated experiments both in *Aldrovanda* and *U. australis* failed to estimate any positive K^+^ uptake from diluted media in light (Adamec, unpublished) and indicated that K^+^ uptake could occur only at high photosynthetic rate or rapid apical shoot growth.

*Utricularia* suction traps are hermetically closed bladders filled by a fluid and function on the basis of negative pressure (Juniper et al., 1989; Adamec, 2011a,b). In addition to their trapping animal prey, commensal microorganisms (bacteria, algae, ciliates, and rotifers) propagate in the traps of aquatic *Utricularia* species. The question of their role in trap functioning and plant nutrition is often discussed. In traps with captured prey, commensals evidently participate in prey digestion by producing their own enzymes (Adamec, 2011a). However, the majority of traps are known to capture no prey during their life-span though their commensal communities are very prolific and dense. To support the communities, traps even exude organic substances into the trap fluid (Sirová et al., 2010). Nevertheless, in barren waters, commensal communities in traps seemed to be more beneficial for the plants than the trapping of prey alone (Richards, 2001). In prey-free traps, which can suck in much detritus or phytoplankton from the ambient water during incidental firings, a miniature microbial food web may run (Sirová et al., 2009) and many *Utricularia* species may rather be considered “bacterivorous” or “detritivorous” than carnivorous. However, unlike the postulated hypothesis on the nutritive role of trap commensals for N and P uptake by traps (Richards, 2001), very high concentrations of total soluble N and P were found in the *Utricularia* trap fluid; the concentrations increased with the trap age and correlated with commensal biomass (Sirová et al., 2009).

To reveal whether *Utricularia* traps can gain N and P from the accumulated organic material inside traps or whether traps rather exude these nutrients for the microbial community, an ecological model based on literature data was devised (Adamec, 2011b). Simply, a theoretical N and P input rate from the natural ambient water into the trap was compared with the estimated total N and P content inside the trap. The model shows that the total N and P content inside the traps is too high to be accumulated from only the ambient water (Adamec, 2011b). Thus, such a low N and P gain cannot be ecologically important for the plant mineral nutrition. Moreover, the prey-free traps do not take up any N and P from the trap fluid but rather exude an amount of N and P to the fluid to support the microbial community. This implies that the uptake affinity of prey-free traps for mineral and organic nutrients is surprisingly very low—unlike the above very high nutrient uptake affinity of shoots—although traps are nutrient absorbing organs. Therefore, the trap microorganisms behave more as parasites than commensals and represent an additional ecological cost for trap maintenance. It is a question, however, whether the nutrient uptake affinity is different in prey-free traps and in those with captured prey. *Utricularia* traps are the most sophisticated ones among all CPs. In spite of their demanding physiological functions, including mineral nutrient uptake from prey, their nutrient absorption affinity is paradoxically very low though the uptake rate in traps with captured prey may be very high. On the contrary, mineral nutrient uptake (N, P, and K) by shoots of aquatic CPs runs with very high affinity but the uptake rate per unit biomass is presumably very low.

In conclusion, thanks to the dynamically growing knowledge of CPs, we are increasingly more able to discuss to what extent CPs are different from or similar to “normal” non-CPs.
